# Generation of bivalent chromatin domains during cell fate decisions

**DOI:** 10.1186/1756-8935-4-9

**Published:** 2011-06-06

**Authors:** Marco De Gobbi, David Garrick, Magnus Lynch, Douglas Vernimmen, Jim R Hughes, Nicolas Goardon, Sidinh Luc, Karen M Lower, Jacqueline A Sloane-Stanley, Cristina Pina, Shamit Soneji, Raffaele Renella, Tariq Enver, Stephen Taylor, Sten Eirik W Jacobsen, Paresh Vyas, Richard J Gibbons, Douglas R Higgs

**Affiliations:** 1 MRC Molecular Haematology Unit, Weatherall Institute of Molecular Medicine, University of Oxford, Oxford, OX3 9DS, UK; 2 Haemopoietic Stem Cell Laboratory, Weatherall Institute of Molecular Medicine, University of Oxford, Oxford, OX3 9DS, UK; 3 Computational Biology Research Group (CBRG), University of Oxford, Oxford, UK; 4 Department of Haematology, University of Oxford, John Radcliffe Hospital, Oxford, OX3 9DS, UK

## Abstract

**Background:**

In self-renewing, pluripotent cells, bivalent chromatin modification is thought to silence (H3K27me3) lineage control genes while 'poising' (H3K4me3) them for subsequent activation during differentiation, implying an important role for epigenetic modification in directing cell fate decisions. However, rather than representing an equivalently balanced epigenetic mark, the patterns and levels of histone modifications at bivalent genes can vary widely and the criteria for identifying this chromatin signature are poorly defined.

**Results:**

Here, we initially show how chromatin status alters during lineage commitment and differentiation at a single well characterised bivalent locus. In addition we have determined how chromatin modifications at this locus change with gene expression in both ensemble and single cell analyses. We also show, on a global scale, how mRNA expression may be reflected in the ratio of H3K4me3/H3K27me3.

**Conclusions:**

While truly 'poised' bivalently modified genes may exist, the original hypothesis that all bivalent genes are epigenetically premarked for subsequent expression might be oversimplistic. In fact, from the data presented in the present work, it is equally possible that many genes that appear to be bivalent in pluripotent and multipotent cells may simply be stochastically expressed at low levels in the process of multilineage priming. Although both situations could be considered to be forms of 'poising', the underlying mechanisms and the associated implications are clearly different.

## Background

In recent years it has been suggested that the epigenetic programme may play a key role in determining cell fate, including the decision to undergo self-renewal or commitment. Based on genome-wide chromatin immunoprecipitation (ChIP) studies combined with expression analysis, it has been suggested that the chromatin associated with many genes controlling lineage fate decisions is uniquely marked in stem cells. Their histone signature is referred to as bivalent as it includes modifications associated both with repression (H3K27me3) imposed by the polycomb group proteins (PcG), and activation (H3K4me3) encoded by the Set/MLL histone methyltransferase, the mammalian homologue of the trithorax group proteins (trxG) [1-6]. Despite having both 'active' and 'repressive' chromatin marks, such genes were thought not to be expressed. Taken together, these observations led to an attractive model suggesting that a preimposed epigenetic signature suppresses expression of lineage control genes in stem cells (maintaining a pluripotent state) while at the same time 'poising' such genes for subsequent activation (reviewed in [7]). In favour of this, many lineage-control genes have a bivalent signature [1-5]. However, as the model has evolved, more recently it has been shown that RNA polymerase II (PolII) may be present but stalled at the promoters of bivalent genes [8,9] and that short (abortive) transcripts may be detected at their promoters [10]. Furthermore, although embryonic stem cells (ES cells) lacking the PcG repressive complex 2 (PRC2) aberrantly express developmental regulators [11] they maintain pluripotency [12]. Similarly, two recent experiments in which components of the SET1/MLL core subunit (Dpy-30, RbBP5 and WDR5) were reduced to similar levels showed opposite phenotypes. In one study there was maintenance of self-renewal with a defect in differentiation [13], and in another there was a loss of self-renewal [14]. Together these observations suggest that the current models explaining the significance bivalently marked chromatin may require revision.

An understated problem in testing the prevailing bivalent chromatin hypothesis is that the criteria for identifying such signatures are poorly defined. Closer analysis of publicly available chromatin datasets from human embryonic stem (ES) cells shows that even contiguous bivalent chromatin domains can be modified in widely different ways with respect to the relative levels and the distributions of H3K4me3 and H3K27me3 across the locus (see Additional file 1). Sequential ChIP analyses of a few developmental genes have shown that H3K4me3 and H3K27me3 may colocalise and, by extrapolation, it has been implied that all genes whose promoters are marked (to any degree) by both modifications are truly bivalent. According to this paradigm, many specialised, lineage-specific genes are bivalent [3,4,15] and as cells differentiate, chromatin modifications resolve into active or repressed states. However, since the original observations, it has become clear that bivalent chromatin modifications (indistinguishable from those seen in pluripotent cells) can also be newly established and/or maintained in differentiating cells [16-19]. Therefore, the functional significance of such bivalently marked genes has been questioned [20] and more studies have been urged to determine the mechanisms underlying these chromatin structures [19].

**Figure 1:**
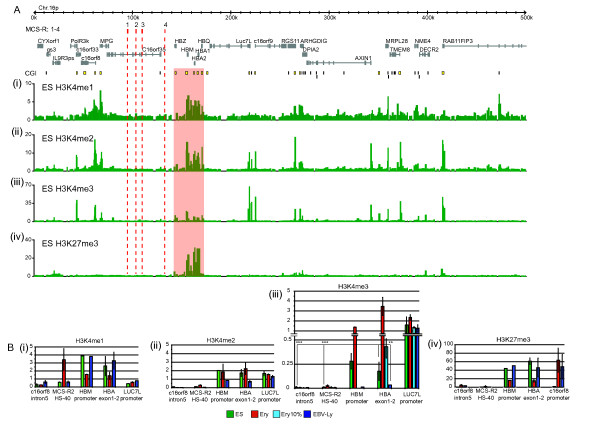
**Chromatin marks at the telomeric region of chromosome 16p in embryonic stem (ES) cells. (a) **The genes, multispecies conserved regulatory sequences (MCS-R1-4) and CpG islands (CGI) are shown at the top. The red shaded box represents the α globin cluster. The y axis represents the enrichment of chromatin immunoprecipitation (ChIP) DNA over input DNA. **(b) **ChIP quantitative PCR (qPCR) data at selected amplicons. Ery = primary human erythroblasts; Ery10% = mixed population consisting of 10% erythroblasts and 90% Ly; ES = human ES cells; Ly = Epstein-Barr virus (EBV)-transformed lymphoblastoid cell line. The fold enrichment has been calculated relative to a control sequence in the *ACTB *promoter. The error bars show the standard deviation of three independent experiments. **P < 0.05; ***P < 0.01 (both by Student's t test).

The human α globin genes are located within a well characterised multigene cluster whose analysis has elucidated many of the general principles underlying the transcriptional and epigenetic regulation of mammalian gene expression. The α globin cluster (5'-HBZ-HBM-HBA2-HBA1-HBQ-3') provides unequivocal examples of specialised, tissue-specific genes consistently scored as bivalent in ES cells [4,5] (Figure 1). Their fully activated expression depends on one or more of four remote conserved regulatory elements (MCS-R1 to R4) [21], which interact with their promoters in erythroid cells via a looping mechanism [22]. Although expressed in a strictly tissue-specific and developmental-stage-specific manner during erythropoiesis, the α globin promoters and much of the body of the associated genes lie within unmethylated CpG islands [23]. Previously, we have described many aspects of the transcription factor and epigenetic programmes associated with hematopoiesis and how they are played out on the α globin cluster [24-27]. Here, we have used this model to investigate in detail the relationship between chromatin marks and mRNA expression during commitment and differentiation into erythroid cells. It appears that, rather than carrying a preimposed bivalent epigenetic signature which silences them, in pluripotent ES cells the α globin genes are repressed by PcG and comodified at readily detectable levels by H3K4me3 when expressed, even at basal levels. To ensure that characterisation of the α globin genes is revealing a general principle that could be relevant to other bivalent genes, we performed global analysis of H3K4me3/H3K27me3 modification and gene expression. The results suggest that our understanding of chromatin bivalency at the α globin locus may explain similar marks found at many other bivalent genes both in pluripotent and differentiating cells and highlights an alternative mechanism for generating bivalent domains.

## Results and discussion

### H3K4me3 at the α globin genes occurs at a low but significant level in ES cells and increases during erythroid differentiation

To dissect the mechanism(s) underlying epigenetic 'bivalency' in ES cells, we studied histone modifications across the telomeric region of chromosome 16 containing the human α globin locus. We found that, whereas H3K4me1 and H3K4me2 were both enriched at the α globin locus (Figure 1a, i and 1ii), H3K4me3 (considered to be a sensitive mark of recent or ongoing transcriptional activity) was relatively low (Figure 1a, iii). However, quantitative real time PCR (qPCR) (Figure 1b, iii) showed that the level of H3K4me3 at the α globin promoter in ES cells was significantly higher than in lymphocytes in which the α globin genes are considered to be fully repressed (see below). Similar results were obtained at the promoter of the HBM gene, a minor α globin-like gene whose promoter is also associated with a large CpG island [28].

To quantify this low level of H3K4me3, we compared the degree of enrichment seen in pluripotent ES cells to that seen in an artificially mixed population of erythroid and non-erythroid cells. This showed (Figure 1b, iii) that the H3K4me3 enrichment seen in ES cells at the α globin gene was even less than that obtained in a mixed population of cells consisting of 10% erythroid cells (presumably fully modified by H3K4me3) (Figure 1b, iii) and 90% lymphocytes (unmodified by H3K4me3, Figure 1b, iii).

These results show that the chromatin associated with the α globin genes is modified at a significant but low level by H3K4me3 in chromatin derived from a population of pluripotent stem cells. However, the level of H3K4me3 modification increases dramatically (15-fold) as cells differentiate into erythroid cells (Figure 1b, iii). This phenomenon is common to many other genes (previously noted to be bivalent in ES cells), which are expressed at high levels late in erythroid differentiation (for example, BLVRB, FAM83F, MTSS1, TNXB) [18]. Conversely, H3K4me3 is barely detectable in lymphocytes in which the α globin genes are repressed.

### H3K27me3 at the α globin genes occurs at high levels in ES cells and decreases during erythroid differentiation

We have previously shown that α globin expression is repressed in non-erythroid cells by PcG and its associated silencing mark H3K27me3. This repression is put in place early in development and then is either reduced in the erythroid lineage or maintained in non-globin-expressing cell types [27].

Here, we extended these observations to determine the pattern of H3K27me3 in pluripotent cells across the telomeric 500 kb of chromosome 16p. As noted in several non-erythroid differentiated cells [27], H3K27me3 enrichment extended across a broad region of the α globin cluster in ES cells (Figure 1a, iv).

Of importance, we next determined the relative levels of H3K27me3 enrichment at the HBM and α globin promoters in ES cells and lymphocytes in which the α globin genes are repressed. ChIP-chip results (Figure 1, iv and [27]), in accordance with qPCR data (Figure 1b, iv), indicate that the locus is modified to a similar extent in both cell types. These findings suggest that the α globin genes are highly (possibly maximally) modified by H3K27me3 in pluripotent cells and that this level of modification is maintained when cells differentiate into non-erythroid lineages. By contrast, in the erythroid population, as the PcG is completely cleared [27], H3K27me3 is reduced fourfold compared to that seen in non-erythroid cells (Figure 1b, iv) but not totally removed.

### The α globin locus is bivalently modified in ES cells

Chromatin modification at the α globin locus thus resembles that seen at other bivalent domains (for example, *CDX2 *(see Additional file 1)) at which there is a high level of H3K27me3, and a low level of H3K4me3 which increases (with expression) or decreases (with silencing) in specific lineages as cells differentiate. To determine if the observed bivalent architecture results from colocalisation of H3K27me3 and H3K4me3 rather than simply reflecting the presence of two distinct subpopulations of active and silent cells, we performed sequential ChIP analyses.

**Figure 2:**
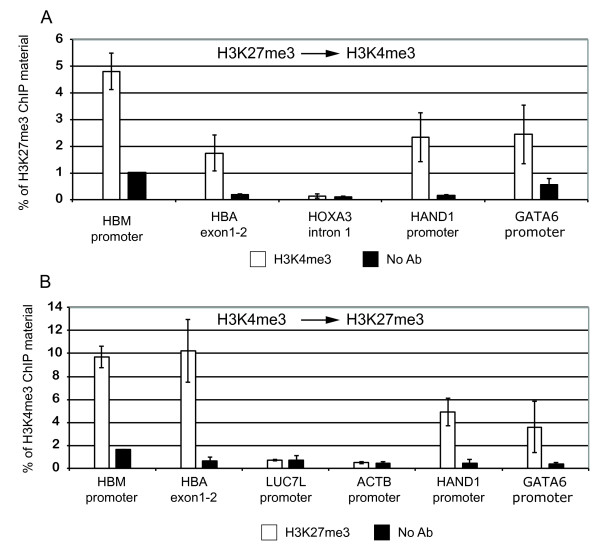
**Chromatin immunoprecipitation quantitative PCR (ChIP-qPCR) characterisation of the α globin chromatin bivalency. (a) **Sequential ChIP with H3K27me3 followed by H3K4me3 antibody. *HOXA3 *intron represents an H3K27me3 genomic region; *HAND1 *and *GATA6 *promoters are control bivalent promoters. **(b) **Sequential ChIP with H3K4me3 followed by H3K27me3 antibody. *LUC7L *and *ACTB *promoter are H3K4me3 modified promoters. The error bars show the standard deviation of three independent ChIP-qPCR experiments.

At α globin and HBM promoters, chromatin precipitated with an antibody against H3K27me3 was sequentially precipitated by an antibody against H3K4me3 (Figure 2a). Similar results were seen by the 'reverse' sequential ChIP (Figure 2b). This shows that at least some chromatin at the α globin genes is truly comodified by H3K27me3 and H3K4me3.

### The α globin locus is expressed at basal levels in a significant proportion of pluripotent cells

Chromatin modification may influence the probability that a locus is transcribed and/or reflect its recent transcriptional state. As for many bivalent genes, the assertion that the α globin genes are not expressed in pluripotent cells is based on the failure to identify binding of PolII in ChIP experiments and the very low (background) signals obtained on RNA microarray analyses. However, using more sensitive assays it has become increasingly clear that in pluripotent cells many bivalent genes are being transcribed either to produce a variety of short, abortive transcripts [8,10] or to produce very low levels of full length RNA transcripts [15,29].

**Figure 3:**
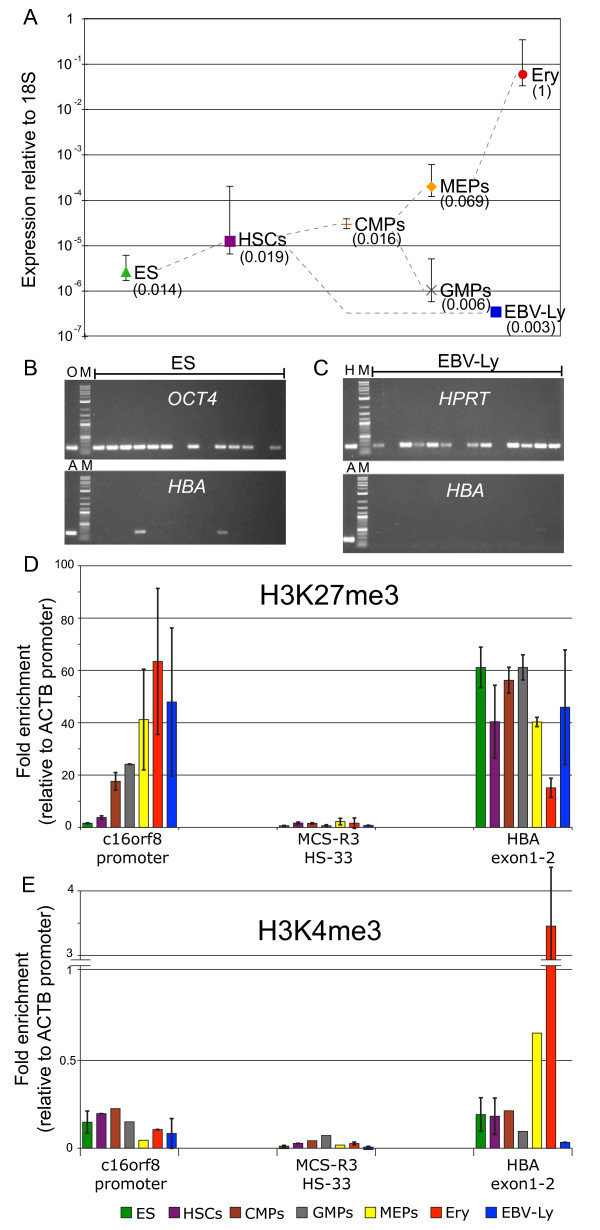
**α Globin expression and chromatin structure in embryonic stem (ES) cells, hematopoietic progenitors and differentiated cells. (a) **Relative expression level of α globin. The error bars represent the standard deviation of three independent experiments. CMPs = common myeloid progenitors; EBV-Ly = Epstein-Barr virus (EBV)-transformed lymphoblastoid cell line; ES = ES cells; Ery = primary erythroblasts; GMPs = granulocyte-monocyte progenitors; HSCs = hematopoietic stem cells; MEPs = megakaryocyte-erythroid progenitors. The ratio between the level of H3K4me3 and H3K27me3, as measured by quantitative PCR (qPCR) at the α globin gene (see below), is shown by each of the cell types analysed. The value obtained in primary erythroblasts was set to 1. **(b) **Examples of *OCT4 *and α globin expression analysis carried out in 270 single ES cells. Each lane corresponds to the same ES single cell. A = α globin control; O = *OCT4 *control; M = molecular weight marker. **(c) **Example of *HPRT *and α globin expression analysis carried out in 110 single EBV-Ly cells. Each lane corresponds to the same EBV-Ly single cell. H = *HPRT *control. Only *OCT4 *or *HPRT *positive cells were considered as informative. **(d) **H3K27me3 chromatin immunoprecipitation (ChIP)-qPCR in hematopoietic progenitors. The fold enrichment is relative to a control sequence in the *ACTB *promoter. The error bars show the standard deviation of two independent experiments. **(e) **H3K4me3 ChIP-qPCR in hematopoietic progenitors. The fold enrichment is relative to a control sequence in the *ACTB *promoter. The error bars show the standard deviation of two independent experiments.

To determine the transcriptional status of the α globin bivalent domain, we firstly looked for, but could not detect, high levels of 5' abortive transcripts (see Additional file 2). Then, we analysed expression of spliced transcripts at the cell population level. We found that in ES cells the level of α globin mRNA, although very low compared to erythroblasts (approximately 30,000 to 40,000 times less), was at least 10 times higher than that measured in Epstein-Barr virus (EBV)-transformed lymphoblastoid (EBV-Ly) cell lines (Figure 3a). Similarly, HBM mRNA could be detected in ES cells (although 50 times less than α globin) but was not detectable in lymphoblastoid cells (see Additional file 2). The lower expression of HBM than α globin might be due to differences in promoter sequences or in mRNA stability of this minor globin gene [28]. By comparison, no β globin RNA transcripts were detected in ES and lymphoblastoid cells. We next estimated how many ES cells within each population express α globin using single-cell RT-PCR with primers that detect full-length mRNA transcripts. Using a multiplex analysis, this showed a detectable level of α globin in 12% of *OCT4 *positive ES cells (Figure 3b). Since a single normal erythroid cell contains approximately 20,000 (α + β) globin RNA molecules [30], it can be estimated that pluripotent ES cells express α globin only at basal (two or three copies per cell) levels. This is consistent with observations that chromatin modifications (H3K36me3 and H3K9me3) associated with high rates of transcription were not detected in ES cells (see Additional file 3). By contrast, no α globin expression was detected in more than 100 EBV-Ly cells (Figure 3c), confirming that in these cells, unlike ES cells, the state of chromatin at the α globin locus is in a completely repressed transcriptional configuration.

### Changes in expression and chromatin modification in hematopoietic stem cells and progenitors

To examine how α globin expression changes during hematopoiesis, we studied primary hematopoietic progenitors at various stages of commitment and differentiation (Figure 3a). Mixed populations enriched for hemopoietic stem cells (HSCs) and common myeloid progenitors (CMPs) exhibited a level of α globin gene expression higher than in ES cells. Expression then increased further in fractions enriched for megakaryocyte-erythroid progenitors (MEPs) but decreased in granulocyte-monocyte progenitors (GMPs), confirming that a gradual restriction in the differentiation potential is associated with an upregulation of lineage-specific genes and silencing of lineage-inappropriate genes.

In HSCs we detected high levels of H3K27me3 and relatively low levels of H3K4me3 (Figure 3d); a pattern consistent with the accepted criteria for a bivalent chromatin signature (and as defined for this gene in pluripotent ES cells). The levels of H3K27me3 (and its associated methyltransferase EZH2) in HSCs, CMPs, GMPs, MEPs and EBV-Ly cells were similar and decreased as MEPs differentiated into erythroid cells (Figure 3d and see Additional file 4). By contrast the levels of H3K4me3, associated with the parallel increase of expression, started to increase as CMPs differentiated into MEPs, prior to the clearance of PcG and H3K27me3. Therefore, at each of these stages of differentiation, the ratio of H3K4me3/H3K27me3 changed, reflecting the level of α globin expression (Figure 3a).

### Bivalent genes with a higher H3K4me3 occupancy at the promoter are more often transcribed in ES cells

In genome-wide analyses it has been shown that, in general, the level of H3K4me3 modification correlates with gene expression and H3K27me3 correlates with silencing [16,17,31,32]. But does this correlation also apply to bivalent genes? The detailed analysis of a single gene presented here shows that, at the bivalent α globin locus, changes in the levels of basal gene expression are reflected by changes in the H3K4me3 mark. Is this behaviour an exception? Here we evaluated the relationship between H3K4me3/H3K27me3 modifications and gene expression at other, previously identified bivalent genes.

**Figure 4:**
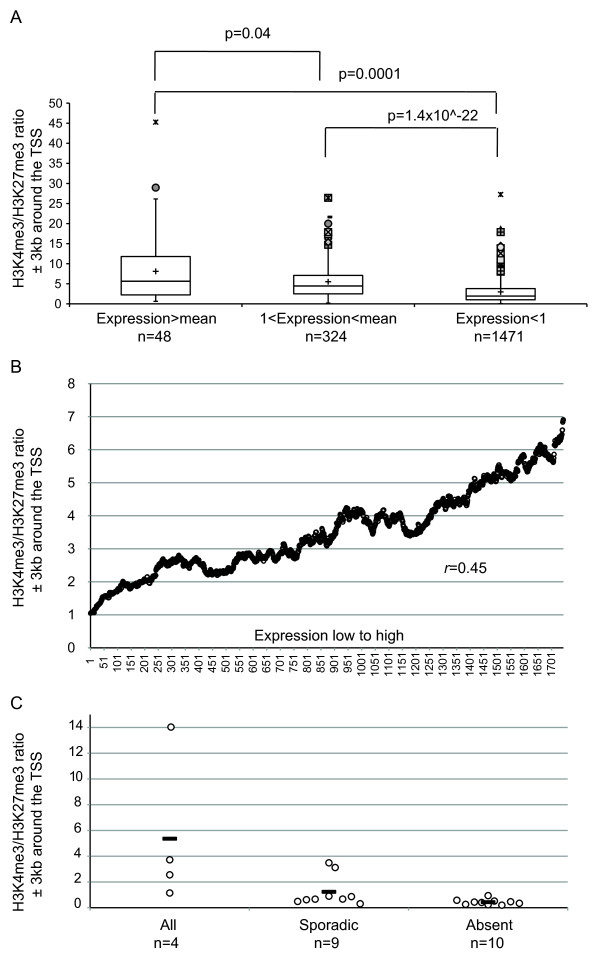
**Correlation between expression level and H3K4me3/H3K27me3 occupancy at bivalent genes in embryonic stem (ES) cells**. **(a) **Box plot showing 25th, 50th and 75th percentiles of H3K4me3/H3K27me3 ratio for 1,843 bivalent genes associated with 3 different expression levels, as calculated by Pan *et al*. [4] (above the mean value for all expressed genes; between the mean value and 1; and below 1). The error bars represent 1.5 times the interquartile range above and below the median. Outliers are plotted individually. The mean of each population is shown as a black cross. Statistical differences were determined using a two-tailed unpaired Student's t test. **(b) **A total of 1,844 bivalent genes were ranked according to their levels of expression (low to high, x axis) and plotted against the moving average (window size 100 genes, step 1) of the ratios of H3K4me3/H3K27me3 (y axis). Spearman's *r *value is shown in the graph. **(c) **Scatterplot showing the H3K4me3/H3K27me3 ratio for 23 bivalent genes associated with 3 different expression patterns as shown by Gibson *et al*. [29] (present in all the cells analysed, present sporadically in some but not all cells, and absent). The mean of each population is shown as a black bar. The H3K4me3/H3K27me3 ratio has been calculated from the signal derived from the number of H3K4me3 and H3K27me3 sequence reads in an area spanning from -3 kb to +3 kb around the transcription start site (TSS). Statistical analysis made with different windows (from -4 kb to +1 kb and from -0.5 kb to +2 kb) gave similar results.

On a global scale, we determined how the H3K4me3/H3K27me3 ratio differed at bivalent genes known to be expressed at different levels in ES cells. Data from human ES cell studies [4,6] were crossreferenced and the bivalent genes subdivided into three 'bins' according to their expression. We found that in a population of ES cells, the chromatin associated with genes with the highest expression had the highest H3K4me3/H3K27me3 ratios, while those with the lowest relative expression had the lowest ratios (Figure 4a). In addition, there was a significant positive association between the absolute level of RNA expression and the H3K4me3/H3K27me3 ratio at the associated transcription start sites (TSSs) (*P *= 2.2 × 10^-16^) (Figure 4b). We also analysed the same data set plotting for each individual bivalent gene the ratio of H3K4me3/H3K27me3 against expression. Clearly they follow the same trend but, due both to biological variation and insensitivity of microarray data to low levels of transcription, there is a considerable scatter (see Additional file 5).

In a cell population, the relative levels of H3K4me3/H3K27me3 at previously identified bivalent genes could reflect the relative proportions of cells in which the gene in question is marked exclusively by H3K4me3 or H3K27me3. To address this we looked at a subset of the previously studied bivalent loci with the highest levels of H3K27me3 (top 20%). It seemed likely that, for this set, the genes in question would be modified by H3K27me3 in most, if not all, cells. So it was of interest that, even in this subgroup, we found a significant positive association between the absolute levels of RNA expression and the levels of associated H3K4me3 (*P *= 2.25 × 10^-8^) (see Additional file 6). Therefore, in comparison to other more stringent forms of silencing (for example DNA methylation), PcG silencing may be incomplete and allow stochastic gene expression.

Finally, we examined the relationship between the ratio of H3K4me3/H3K27me3 at specific bivalent genes and the ability to detect single-cell transcripts expressed from these genes. It has been reported that, as for α globin, expression of other bivalent genes can be sporadically detected in single ES cells [29]. The bivalent genes studied by Gibson *et al*. [29] were crossreferenced with ChIP data from Ku *et al*. [6] and subdivided into three classes according to the proportion of cells in which the transcript was detected. Although the number of genes analysed by single-cell RT-PCR was small, we found that there was a clear tendency for the genes whose transcripts were never detected, to have the lowest H3K4me3/H3K27me3 ratios (Figure 4c).

## Conclusions

The central question raised here is whether or not bivalent domains, marked both by repressive (H3K27me3) and active (H3K4me3) histone modifications, represent a preprogrammed epigenetic signature of silent but poised chromatin or more simply reflect different degrees of silencing (H3K27me3) and transcription (H3K4me3) within a population of cells. The observation that most clearly supports the prevailing concept of a preprogrammed epigenetic mark in pluripotent cells is the presence of bivalent domains in which, despite the presence of both activating (H3K4me3) and repressive (H3K27me3) marks, no PolII elongation and full length transcription are detected, leading to the conclusion that all of such genes are poised [8,9]. However through evaluation of public datasets it is readily notable that the levels and patterns of histone modifications at bivalent genes are very variable. Similarly, a wide range of H3K4me3 levels within bivalent domains of embryonic and haematopoietic stem and progenitor cells has been recently identified [13,19].

Here, the detailed analysis of a single well characterised bivalent gene (α globin) expressed during haematopoiesis and the global analysis of the relationship between H3K4me3/H3K27me3 ratio and gene expression suggest another explanation for bivalent chromatin domains that might be considered. It is possible that some genes with bivalent signatures may be poised in pluripotent cells by a common but as yet undefined molecular mechanism. If so, then it is not clear why the associated chromatin modifications are so variable. An alternative explanation, proposed here, is that bivalently marked genes are regulated by PcG and marked by H3K27me3; the different levels of associated H3K4me3 may simply be a sensitive marker of different levels of transcriptional activity. The latter scenario is consistent with previous observations showing that many lineage-specific genes can be stochastically expressed at low but variable levels in multipotent cells (so called multilineage priming) [33].

Although both situations could be considered to be poised the underlying mechanisms and their implications are different. In fact, the first model (repressed but poised for later activation) implies that most of the cells harbour coexisting repressive and activating marks and that both PcG and trxG have to be maintained at the given locus through cell divisions to keep the repressed-but-poised status until further changes in the balance of their activity. By contrast, the second (repressed and marked by variable degrees of stochastic transcription) implies that PcG is not fully efficient at repressing stochastic transcriptional noise. This would provide fertile ground for subsequent activation and changes in cell fate decisions as a tissue-specific transcription factor programme emerges [34]. Definitive experiments to discriminate between these two possibilities will require the development of assays to correlate chromatin modification and gene expression within single cells.

## Methods

### Ethics

The part of the study involving human participants was approved by the Institutional Ethics Committee (approval number 06/Q1606/110). A written informed consent was obtained from all patients.

### Primary cells and cell culture

EBV-Ly cell lines were cultured in RPMI 1640 supplemented with 10% (v/v) fetal calf serum, 2 mM l-glutamine, 50 U/ml penicillin and 50 μg/ml streptomycin. Isolation and culture of primary human erythroblasts was carried out as described previously [35]. The human embryonic stem cell line, H1 (WiCell, Madison, WI, USA), was grown on irradiated mouse embryonic fibroblasts in medium containing Dulbecco's modified Eagle medium (DMEM):F12, serum replacer (Invitrogen, Paisley, UK), l-glutamine, β-mercaptoethanol, non-essential amino acids and basic fibroblast growth factor (8 ng/ml). Cells were passaged every 5-7 days with collagenase IV to maintain undifferentiated human embryonic stem cells.

### Flow cytometric analysis and sorting

Normal human bone marrow samples were collected from individuals undergoing total hip replacement for osteoarthritis. CD34+ cells were enriched using MACS (Miltenyi Biotech, Bergisch Gladbach, Germany) immunomagnetic beads and cryopreserved in 90% fetal bovine serum (FBS)/10% dimethylsulfoxide (DMSO). All experiments were carried out using cryopreserved CD34+ cells that were thawed, washed in Iscove's modified Dulbecco's medium (IMDM)/10% FBS and cultured overnight in StemSpanSFEM (StemCell Technologies, Grenoble, France) in the presence of recombinant human stem cell factor (100 ng/ml), Flt3-ligand (100 ng/ml) and thrombopoietin (100 ng/ml) (Peprotech, Rock Hill, NJ, USA).

CD34+ cells were first stained with purified anti-CD2, RPA-2.10; CD3, HIT3a; CD4, RPA-T4; CD7,124-1D1; CD8, RPA-T8; CD10, CB-CALLA; CD11b, ICRF44; CD14, 61D3; CD19, HIB19; CD20, 2H7; CD56, MEM188; GPA, GA-R2 (eBioscience, San Diego, CA, USA). Subsequently cells were stained with Pacific Blue conjugated goat F(ab')_2 _anti-mouse IgG conjugates (H+L) (Invitrogen). Finally, cells were stained with fluorescein isothiocyanate (FITC)-conjugated anti-CD38 (HIT2), phycoerythrin (PE)-conjugated anti-CD45RA (HI100), PE-Cy7-conjugated anti-CD123 (6H6) (eBioscience), PE-Cy5-conjugated anti-CD34 (581) (Beckman Coulter, High Wycombe, UK) and allophycocyanin (APC)-conjugated anti-CD110 (BAH-1) (Becton Dickinson, Franklin Lakes, NJ, USA). HSCs were isolated as Lin- CD34+ CD38-, CMPs as Lin- CD34+ CD38+ CD123low/+ CD45RA- CD110-, GMPs as Lin- CD34+ CD38+ CD123+ CD45RA+ CD110- and MEPs as Lin- CD34+ CD38+ CD123-/low CD45RA- CD110-. Dead cells were excluded by Hoechst 33258 (Invitrogen) staining or by 7-aminoactinomycin D (Sigma-Aldrich, Gillingham, UK). Appropriate unstained, single stained and Fluorescence Minus One controls were used to determine the background staining level and compensation in each channel. All sorting and analyses were performed on three laser-equipped MoFlo (Dako Cytomation, Ely, UK) or BD FACS AriaII (Becton Dickinson) machines. Single ES cells were sorted with an automated cell deposition unit into 96-well plates. FACS data were analysed with Summit software (Dako Cytomation).

### ChIP assays

ChIP analyses were performed according to the Millipore ChIP protocol (Millipore 17-295, Billerica, MA, USA), as described previously [26]. Input and immunoprecipitated material were analysed by real time PCR using a series of PCR amplicons and 5'FAM-3'TAMRA probes across the α globin locus [26]. ChIP-chip experiments were carried out on the custom α globin tiling path microarray, as described previously [22]. The enrichment of ChIP DNA over input DNA was calculated as ratio of the background corrected ChIP signal divided by the background corrected input signal (both globally normalised). The antibodies used in the experiments were: anti-mono/di/trimethyl Lys4 histone H3 (07-436, 07-030, and 07-473, respectively), anti-trimethyl Lys27 histone H3 (07-449) and anti-trimethyl Lys9 histone H3 (07-442 lot 24416) from Millipore; anti-trimethyl Lys36 histone H3 (ab9050) from Abcam (Cambridge, UK); anti-RNA-PolII (H224) from Santa Cruz Biotechnology (Santa Cruz, CA, USA), anti-EZH2 (36-6300) from Zymed (San Francisco, CA, USA).

For ChIP in the artificially mixed population of cells, 10% of primary erythroblasts were mixed with 90% of EBV-ly cells. At any one time, about 80% of erythroid cells are positive for nascent α globin RNA transcripts [36] and 100% of these cells accumulate high levels of globin RNA during the late stages of erythropoiesis. Therefore these cells are presumed to be 100% modified by H3K4me3. By contrast, in lymphocytes, the α globin genes are repressed and the H3K4me3 signal at the α globin promoter is at background level.

Sequential ChIP analyses were performed as follows: 5 μg of H3K4me3 or H3K27me3 antibody were immobilised to 50 μl of protein A agarose beads (Millipore) by crosslinking with 2.5 mM BS3 (Thermo Fisher Scientific, Waltham, MA, USA). Chromatin precipitated using the first antibody was eluted in 500 μl of 0.1 M NaHCO_3 _and 1% SDS. This solution was then diluted tenfold in ChIP Dilution buffer (Millipore) and subjected to immunoprecipitation using the second antibody. The second antibody was not crosslinked to protein A.

### Gene expression analysis

Total RNA was extracted using TriReagent (Sigma). Contaminating DNA was removed from RNA preps with the DNA-free kit (Ambion/Applied Biosystems, Austin, TX, USA) according to the manufacturer's instructions. cDNA was generated using 1-5 μg of total RNA and random primers using the Prostar RT-PCR kit (Stratagene/Agilent Technologies, Santa Clara, CA, USA). Negative control cDNA samples generated without reverse transcriptase were analysed in all experiments. Real time qPCR experiments were carried out on ABI Prism 7000 Sequence Detection System (Applied Biosystems) using a set of primers/probe detecting *HBA2 *cDNAs (see Additional file 7). The results were normalised to a control sequence in the 18S ribosomal RNA (*RNRI*) gene (Eurogentec, Southampton, UK). To detect short abortive transcripts at the 5' region of *HBA *cDNA, two different sets of primers were used in a SybrGreen qPCR reaction (see Additional file 7).

Multiplex single-cell RT-PCR analysis was performed as previously described [33,37]. Single cells were deposited into 96-well PCR plates using a single cell depositor unit coupled to a fluorescence-activated cell sorting (FACS) ARIAII cell sorter (providing single cells in >99% of the wells, and no wells with more than 1 cell as assessed by routinely sorting fluorescent beads or cells prior to and after single cell sorting). Each well contained 4 μl of lysis buffer (0.4% NP40, 2.3 mM dithiothreitol (DTT), 0.07 mM dNTP, 0.5 U/μl Rnase Inhibitor). Cell lysates were reverse transcribed in a 10 μl reaction with Superscript III Reverse Transcriptase (Invitrogen) and gene-specific primers (R1s in Additional file 7). A first round of PCR (40 cycles) was performed by the addition of a PCR mix containing PCR buffer, 1.25 U of Taq polymerase (Invitrogen) and gene-specific forward primers (F1s in Additional file 7). A total of 1 μl of 1:10 diluted first-round PCR products were amplified in a second-round PCR, which was carried out using fully nested gene-specific primers (F2-R2 in Additional file 7). PCR products were gel electrophoresed and visualised by ethidium bromide staining. Only control-positive (*OCT4 *in ES cells, *HPRT *in EBV-Ly) wells were considered as informative and scored. A total of 270 individual human ES cells and 110 EBV-Ly were analysed.

### Statistical analysis

In order to correlate genes expression and chromatin state in human ES cells, expression data (accession number GSE8439 [4]) and single cell transcript detection data [29] were crossreferenced with ChIP-Seq data (accession number GSE13084 [6]). The promoter chromatin state was calculated as relative ratio of the signal derived from the number of H3K4me3 and H3K27me3 sequence reads across a window between -3 kb and +3 kb of the annotated TSS. The relationship between H3K4me3/H3K27me3 ratio and expression was calculated by averaging of the H3K4me3/H3K27me3 ratio within a sliding window 100 observations wide, incrementing by 1, using a Spearman rank correlation. Considering the different source of the two ES cell datasets (H1, male cell line, for expression data and H9, female cell line, for ChIP-Seq data), X-chromosome linked genes were excluded from the analysis.

## Competing interests

The authors declare that they have no competing interests.

## Authors' contributions

MDG participated in the design of the study, carried out the molecular studies and drafted the manuscript; DG, ML, DV, NG, SL, KML, JAS-S, CP and RR carried out the molecular studies; DG, ML and RJG helped to draft the manuscript; JRH and ST participated in the bioinformatic and statistical analyses; SS performed the statistical analysis; SEWJ, PV, RJG and TE participated in the design of the study and its coordination; DHR conceived the study and participated in its design and coordination and drafted the manuscript. All authors read and approved the final manuscript.

## Supplementary Material

Additional file 1 **Example of contiguous bivalent domains differing in the extent of H3K4me3 and H3K27me3**. The level and distribution of H3K4me3 and H3K27me3 at two contiguous bivalent domains (*CDX2 *and *FLT3*) are shown in comparison to a housekeeping gene (*PAN3*), which is only modified by H3K4me3. The profile of the two modifications are from Ku *et al *[6]. Click here for file

Additional file 2 **Expression of fully-spliced and 5'-transcript α globin in embryonic stem (ES) cells and differentiated cells**. Expression level of α globin and HBM transcripts is shown relative to 18S. The error bars represent the standard deviation of three independent experiments. EBV-Ly = Epstein-Barr virus (EBV)-transformed lymphoblastoid cell line; Ery = primary erythroblasts; ES = ES cells. HBA 5' Primer Set 1 and HBA 5' Primer Set 2, both spanning the 5' untranslated region (UTR) and the first exon of α globin, were used in order to detect 5' abortive transcripts. Click here for file

Additional file 3 **H3K36me3 and H3K9me3 marks at the telomeric region of chromosome 16p**. Both of these chromatin marks have been associated with actively transcribed regions [3,38]. The genomic region is annotated as in Figure 1. The y axis represent the enrichment of chromatin immunoprecipitation (ChIP) DNA over input DNA calculated as ratio of the background corrected ChIP signal divided by the background corrected input signal (both globally normalised). The red shaded box represents the α cluster locus. EBV-Ly = Epstein-Barr virus (EBV)-transformed lymphoblastoid cell line; Ery = primary erythroblasts; ES = embryonic stem (ES) cells. Click here for file

Additional file 4 **EZH2 chromatin immunoprecipitation quantitative PCR (ChIP-qPCR) in pluripotent cells, hematopoietic progenitors and differentiated cells**. Real-time qPCR analysis of EZH2 ChIP performed in embryonic stem (ES) cells (ES), hematopoietic stem cells (HSCs), common myeloid progenitors (CMPs), granulocyte-monocyte progenitors (GMPs), megakaryocyte-erythroid progenitors (MEPs), primary erythroblasts (Ery), and Epstein-Barr virus (EBV)-transformed lymphoblastoid cell line (EBV-Ly). C16orf8 promoter is a positive control promoter in which the silencing effect of PcG and H3K27me3 occurs late in differentiation [27]. MCS-R3 is a PcG/H3K27me3 negative amplicon. The error bars show the standard deviation of two independent experiments Click here for file

Additional file 5 **Correlation between expression level and H3K4me3/H3K27me3 occupancy**. The increasing trend in H3K4me3/H3K27me3 ratio (y axis, log2 scale) with respect to expression (x axis, log 10 scale) is shown as an XY scatterplot. Each circle corresponds to a single bivalent gene. A line corresponding to the median expression level (2.43) of all genes expressed in embryonic stem (ES) cells, as from Pan *et al*. [4], is shown. Lines corresponding to 25th, 50th and 75th percentiles of the H3K4me3/H3K27me3 ratio are also shown. Bivalent genes analysed in sequential chromatin immunoprecipitation (ChIP) in the current study are displayed in colour and labelled. α Globin and HBM are shown as red and purple dots, respectively. The 20-period moving average trendline is displayed. Click here for file

Additional file 6 **Correlation between expression level and H3K4me3/H3K27me3 occupancy in the top 369 H3K27me3 modified bivalent genes in embryonic stem (ES) cells**. The increasing trend in H3K4me3/H3K27me3 ratio [6] with respect to expression [4] is shown by averaging within a sliding window 20 observations wide, incrementing by 1. Spearman's *r *value is shown in the graph. Click here for file

Additional file 7 **Primers**. Primers used in expression analysis. Click here for file
