# Supplementary figures and images for: Generation of bivalent chromatin domains during cell fate decisions
Source: Epigenetics Chromatin. 2011 Jun 6;4:9. doi: 10.1186/1756-8935-4-9 (PMC3131236; doi:10.1186/1756-8935-4-9)

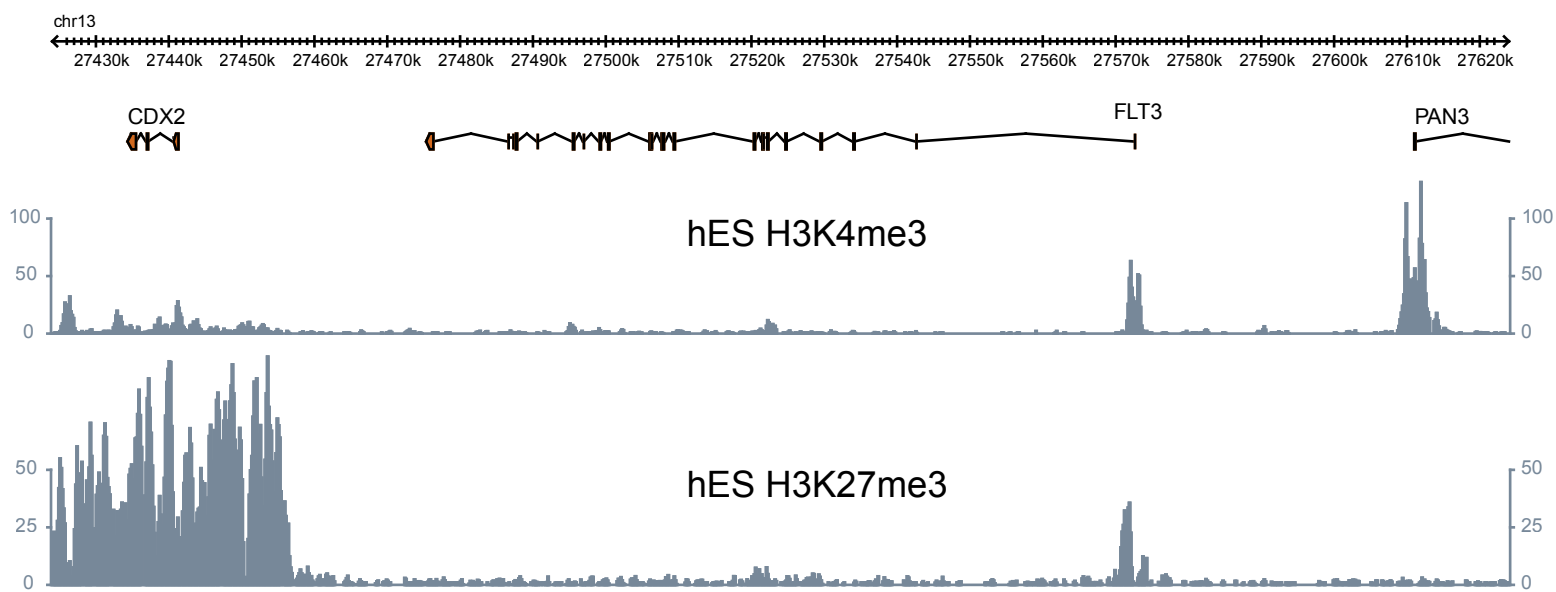

Supplement: Additional file 1 — Example of contiguous bivalent domains differing in the extent of H3K4me3 and H3K27me3. The level and distribution of H3K4me3 and H3K27me3 at two contiguous bivalent domains (CDX2 and FLT3) are shown in comparison to a housekeeping gene (PAN3), which is only modified by H3K4me3. The profile of the two modifications are from Ku et al [6]. [file 1756-8935-4-9-S1.PDF]

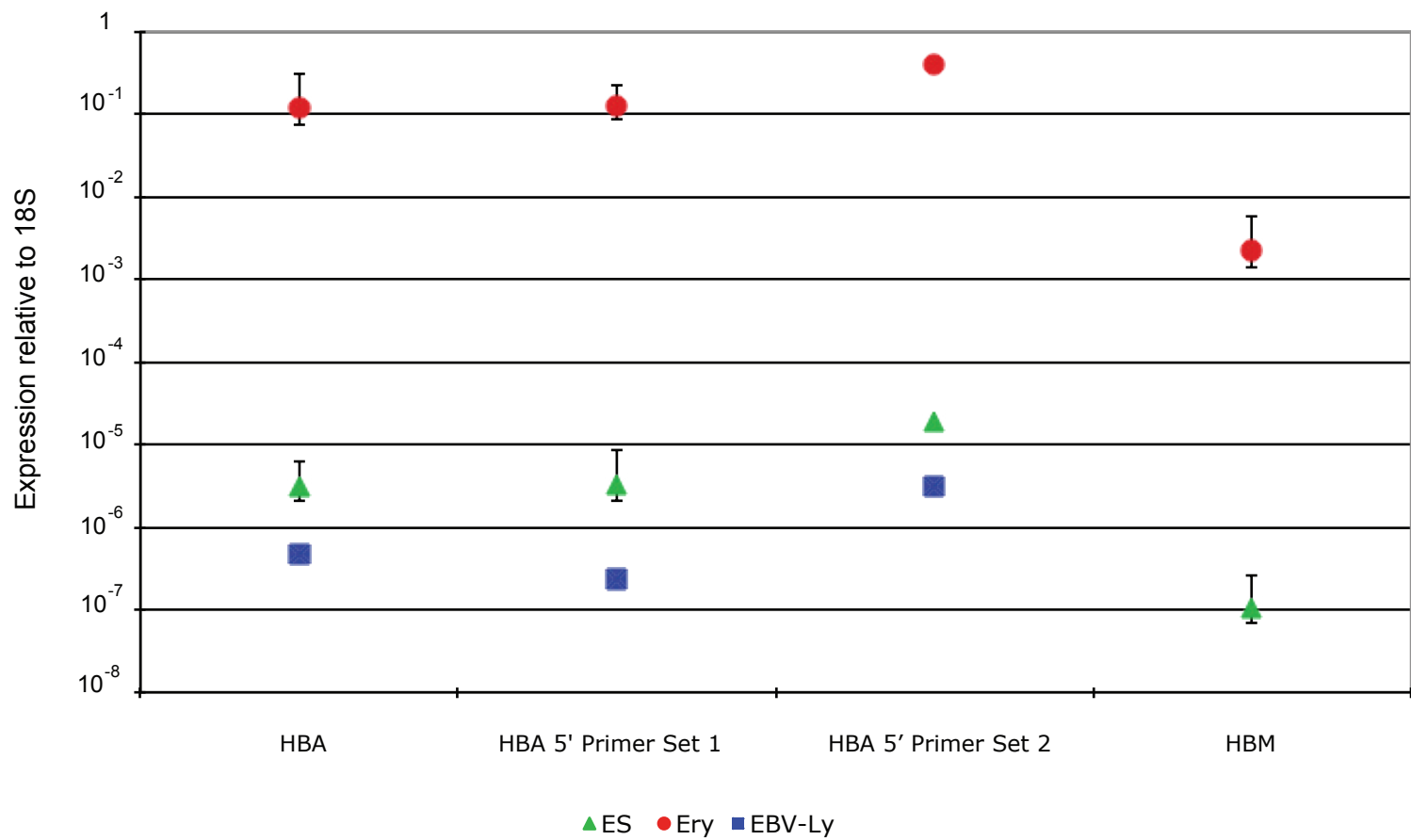

Supplement: Additional file 2 — Expression of fully-spliced and 5'-transcript α globin in embryonic stem (ES) cells and differentiated cells. Expression level of α globin and HBM transcripts is shown relative to 18S. The error bars represent the standard deviation of three independent experiments. EBV-Ly = Epstein-Barr virus (EBV)-transformed lymphoblastoid cell line; Ery = primary erythroblasts; ES = ES cells. HBA 5' Primer Set 1 and HBA 5' Primer Set 2, both spanning the 5' untranslated region (UTR) and the first exon of α globin, were used in order to detect 5' abortive transcripts. [file 1756-8935-4-9-S2.PDF]

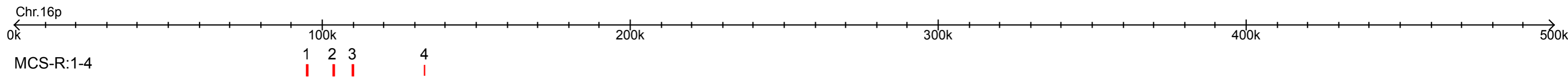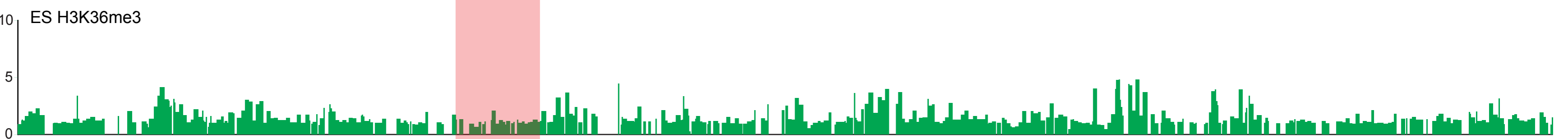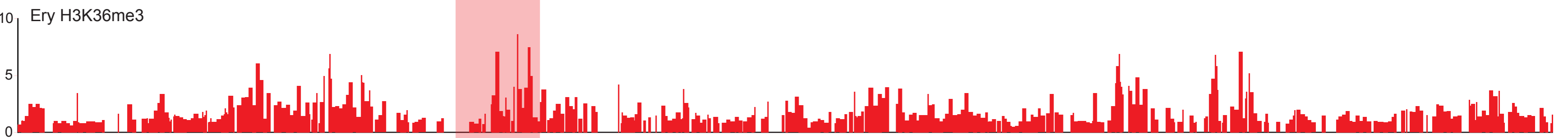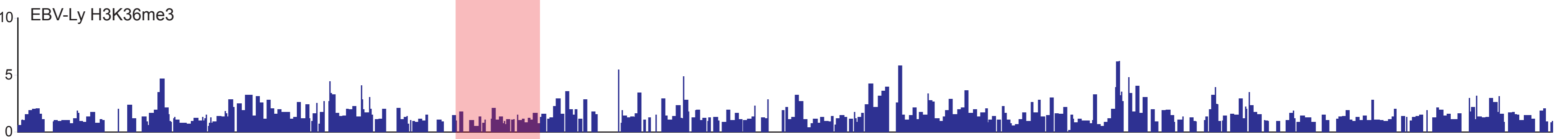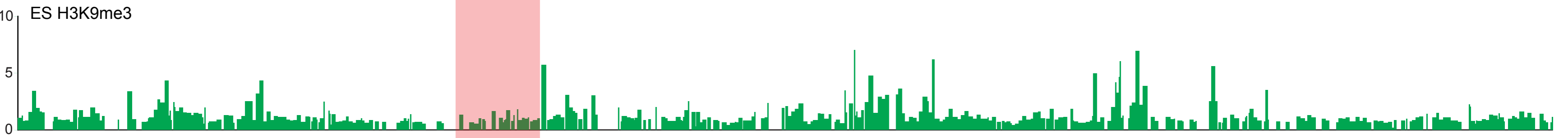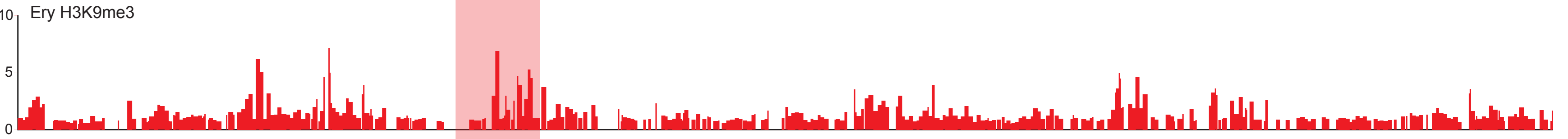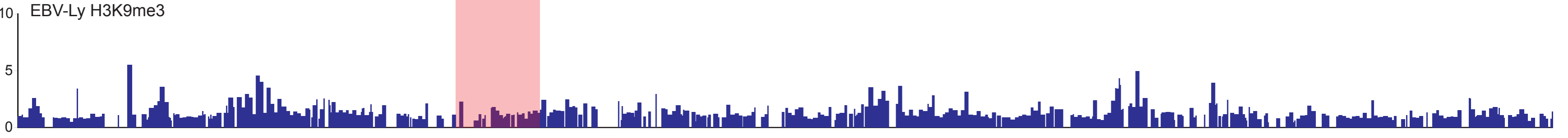

Supplement: Additional file 3 — H3K36me3 and H3K9me3 marks at the telomeric region of chromosome 16p. Both of these chromatin marks have been associated with actively transcribed regions [3,38]. The genomic region is annotated as in Figure 1. The y axis represent the enrichment of chromatin immunoprecipitation (ChIP) DNA over input DNA calculated as ratio of the background corrected ChIP signal divided by the background corrected input signal (both globally normalised). The red shaded box represents the α cluster locus. EBV-Ly = Epstein-Barr virus (EBV)-transformed lymphoblastoid cell line; Ery = primary erythroblasts; ES = embryonic stem (ES) cells. [file 1756-8935-4-9-S3.PDF]

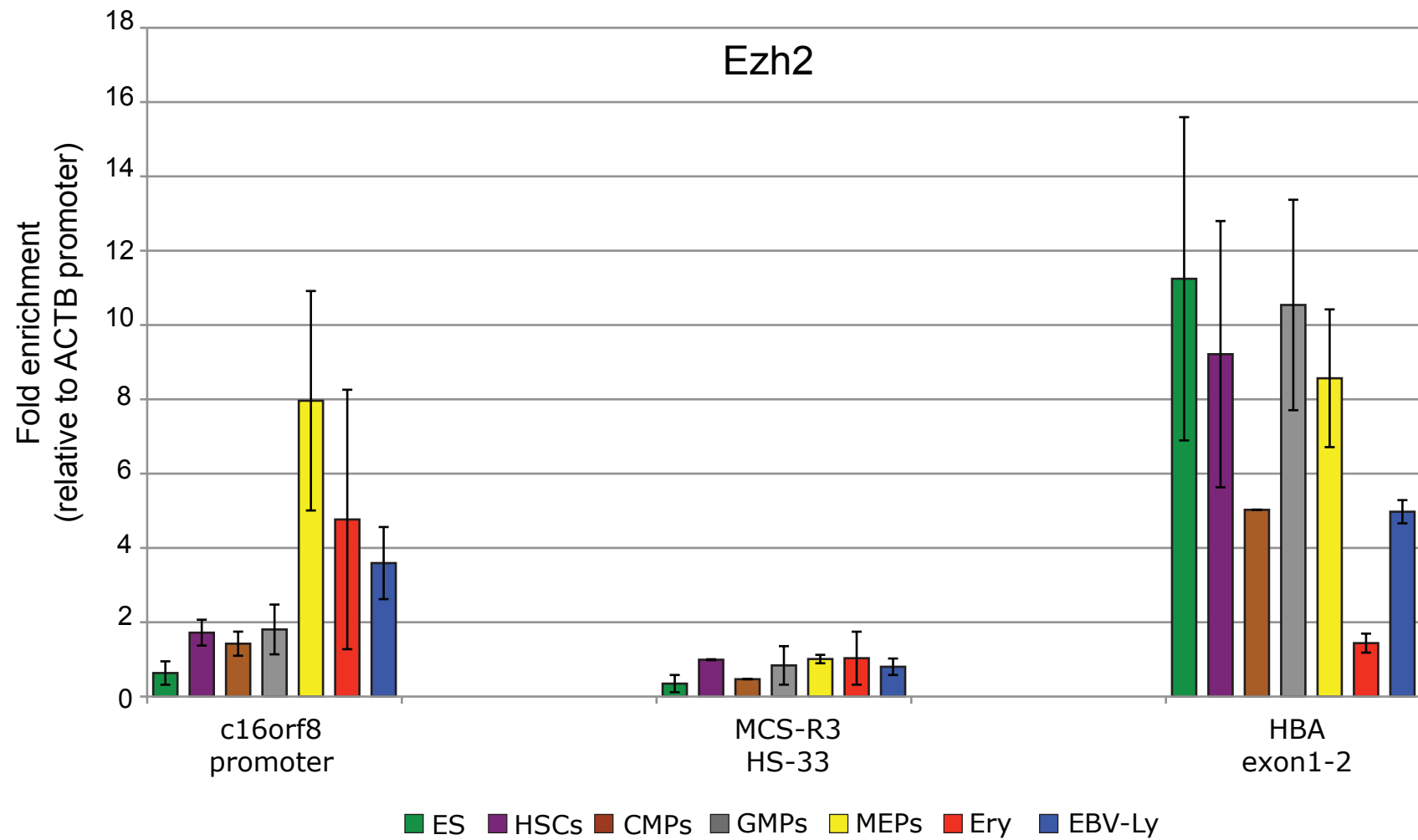

Supplement: Additional file 4 — EZH2 chromatin immunoprecipitation quantitative PCR (ChIP-qPCR) in pluripotent cells, hematopoietic progenitors and differentiated cells. Real-time qPCR analysis of EZH2 ChIP performed in embryonic stem (ES) cells (ES), hematopoietic stem cells (HSCs), common myeloid progenitors (CMPs), granulocyte-monocyte progenitors (GMPs), megakaryocyte-erythroid progenitors (MEPs), primary erythroblasts (Ery), and Epstein-Barr virus (EBV)-transformed lymphoblastoid cell line (EBV-Ly). C16orf8 promoter is a positive control promoter in which the silencing effect of PcG and H3K27me3 occurs late in differentiation [27]. MCS-R3 is a PcG/H3K27me3 negative amplicon. The error bars show the standard deviation of two independent experiments [file 1756-8935-4-9-S4.PDF]

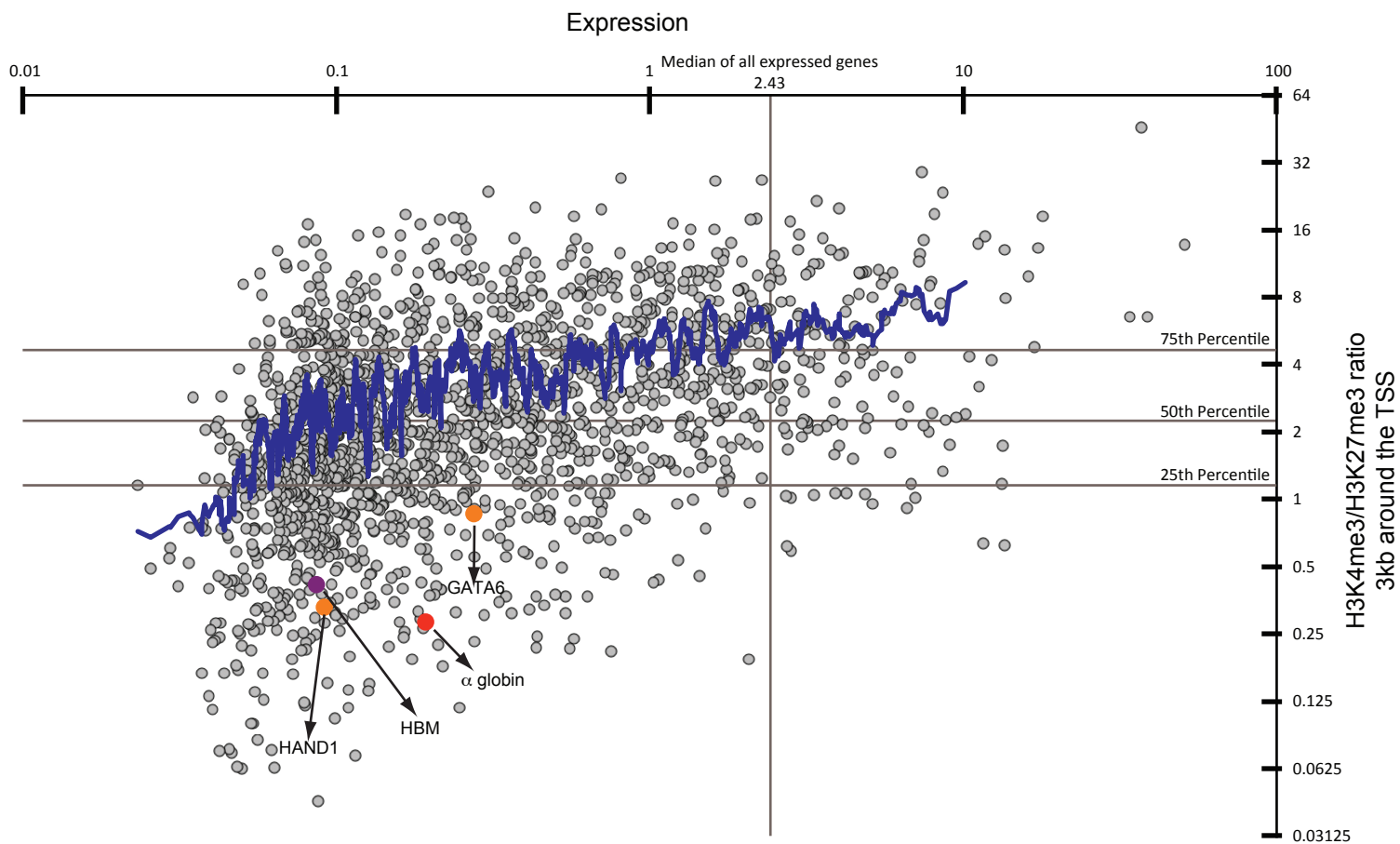

Supplement: Additional file 5 — Correlation between expression level and H3K4me3/H3K27me3 occupancy. The increasing trend in H3K4me3/H3K27me3 ratio (y axis, log2 scale) with respect to expression (x axis, log 10 scale) is shown as an XY scatterplot. Each circle corresponds to a single bivalent gene. A line corresponding to the median expression level (2.43) of all genes expressed in embryonic stem (ES) cells, as from Pan et al. [4], is shown. Lines corresponding to 25th, 50th and 75th percentiles of the H3K4me3/H3K27me3 ratio are also shown. Bivalent genes analysed in sequential chromatin immunoprecipitation (ChIP) in the current study are displayed in colour and labelled. α Globin and HBM are shown as red and purple dots, respectively. The 20-period moving average trendline is displayed. [file 1756-8935-4-9-S5.PDF]

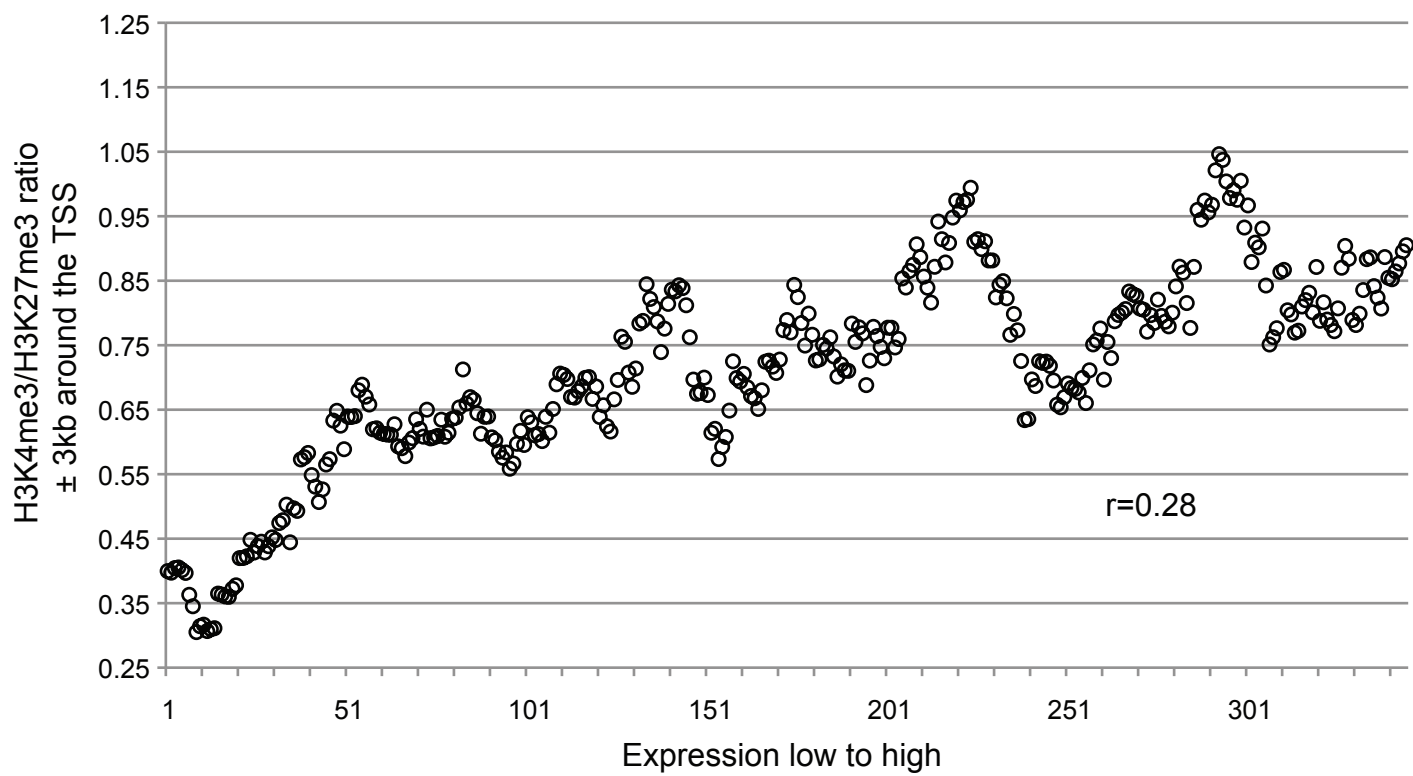

Supplement: Additional file 6 — Correlation between expression level and H3K4me3/H3K27me3 occupancy in the top 369 H3K27me3 modified bivalent genes in embryonic stem (ES) cells. The increasing trend in H3K4me3/H3K27me3 ratio [6] with respect to expression [4] is shown by averaging within a sliding window 20 observations wide, incrementing by 1. Spearman's r value is shown in the graph. [file 1756-8935-4-9-S6.PDF]
