# Supplementary material for: Generation of bivalent chromatin domains during cell fate decisions
Source: Epigenetics Chromatin. 2011 Jun 6;4:9. doi: 10.1186/1756-8935-4-9 (PMC3131236; doi:10.1186/1756-8935-4-9)
Supplement: Additional file 7 — Primers. Primers used in expression analysis. [file 1756-8935-4-9-S7.PDF]

Additional file 7 - Primers used in expression analysis

Cell population RT-PCR	
HBA2-F	GCCCTGGAGAGGATGTTCCT
HBA2-R	CGTGGCTCAGGTCGAAGTG
HBA2-P	5’-FAM-CCTTCCCCACCACCAAGACCTACTTCC-TAMRA-3’
HBA2-5’F1	CTCTTCTGGTCCCCACAGA
HBA2-5’R1	GGCAGGAGACAGCACCAT
HBA2-5’F2	CCCACAGACTCAGAGAGAACCCACCATG
HBA2-5’R2	GGCCTTGACGTTGGTCTTGT
HBM-F	GCGTGGACCCAGCCAACTT
HBM-R	CAGGTGGGAGGCCAGCAC
HBM-P	5’-FAM-TCCGCTGCTAATCCAGTGTTTCCAC-TAMRA-3’
**Single-cell RT-PCR**	
HBA2-F1	TCCCCACAGACTCAGAGAGAACC
HBA2-R1:	GCTCACAGAAGCCAGGAACTTG
HBA2-F2	TGGCGAGTATGGTGCGGAG
HBA2-R2	CAGCAGGCAGTGGCTTAGGAG
OCT4-F1	AGGTATTCAGCCAAACGACCATC
OCT4-R1:	ACCCAGCAGCCTCAAAATCC
OCT4-F2	TGGAGGAAGCTGACAACAATGAAAATC
OCT4-R2	CCGGTTACAGAACCACACTCGGAC;
HPRT-F1	TGATGAAGGAGATGGGAGGC
HPRT-R1	TCCAACACTTCGTGGGGTCC
HPRT-F2	ATTCTTTGCTGACCTGCTGG
HPRT-R2	GCGACCTTGACCATCTTTGG
